# Implementing strategies to prevent infections in acute-care settings

**DOI:** 10.1017/ice.2023.103

**Published:** 2023-07-11

**Authors:** Kavita K. Trivedi, Joshua K. Schaffzin, Valerie M. Deloney, Kathy Aureden, Ruth Carrico, Sylvia Garcia-Houchins, J. Hudson Garrett, Janet Glowicz, Grace M. Lee, Lisa L. Maragakis, Julia Moody, Ann Marie Pettis, Sanjay Saint, Marin L. Schweizer, Deborah S. Yokoe, Sean Berenholtz

**Affiliations:** 1 Alameda County Public Health Department, San Leandro, California; 2 Children’s Hospital of Eastern Ontario, University of Ottawa, Ottawa, Ontario, Canada; 3 Society for Healthcare Epidemiology of America (SHEA), Arlington, Virginia; 4 Advocate Aurora Health, Downers Grove, Illinois; 5 Division of Infectious Diseases, University of Louisville School of Medicine, Louisville, Kentucky; 6 The Joint Commission, Oakbrook Terrace, Illinois; 7 Centers for Disease Control and Prevention, Atlanta, Georgia; 8 Stanford Children’s Health, Stanford, California; 9 Johns Hopkins University School of Medicine, Baltimore, Maryland; 10 Clinical Services Group, HCA Healthcare, Nashville, Tennessee; 11 University of Rochester Medicine, Rochester, New York; 12 VA Ann Arbor Healthcare System and University of Michigan, Ann Arbor, Michigan; 13 University of Wisconsin–Madison, Madison, Wisconsin; 14 University of California San Francisco School of Medicine, UCSF Medical Center, San Francisco, California

## Abstract

This document introduces and explains common implementation concepts and frameworks relevant to healthcare epidemiology and infection prevention and control and can serve as a stand-alone guide or be paired with the “SHEA/IDSA/APIC Compendium of Strategies to Prevent Healthcare-Associated Infections in Acute Care Hospitals: 2022 Updates,” which contain technical implementation guidance for specific healthcare-associated infections. This Compendium article focuses on broad behavioral and socio-adaptive concepts and suggests ways that infection prevention and control teams, healthcare epidemiologists, infection preventionists, and specialty groups may utilize them to deliver high-quality care. Implementation concepts, frameworks, and models can help bridge the “knowing-doing” gap, a term used to describe why practices in healthcare may diverge from those recommended according to evidence. It aims to guide the reader to think about implementation and to find resources suited for a specific setting and circumstances by describing strategies for implementation, including determinants and measurement, as well as the conceptual models and frameworks: 4Es, Behavior Change Wheel, CUSP, European and Mixed Methods, Getting to Outcomes, Model for Improvement, RE-AIM, REP, and Theoretical Domains.

## Intended use

This document introduces and explains common implementation concepts and frameworks relevant to healthcare epidemiology and infection prevention and control. It focuses on broad behavioral and socioadaptive concepts and suggests ways that infection prevention and control teams, healthcare epidemiologists, infection preventionists, and specialty groups may utilize them to deliver high-quality care. This article can be used as a standalone document, or it can be paired with the manuscripts of the “Compendium of Strategies to Prevent Healthcare-Associated Infections in Acute Care Hospitals: 2022 Updates,” which provide technical guidance on how to implement prevention efforts for specific healthcare-associated infections (HAIs).

Implementation concepts, frameworks, and models can help bridge the “knowing–doing” gap, a term used to describe why practices in healthcare may diverge from those recommended according to evidence. It is not comprehensive; it guides the reader to think about implementation and to find resources suited for a specific setting and circumstances.

It also is not intended to be prescriptive. Implementation as a concept is broad, and success in implementing practices or interventions depends on a systematic approach matched to an organization’s context (ie, local factors, such as operational support, informatics resources, experience, willingness to change, safety culture, and others). This guidance and the HAI-specific Compendium articles’ implementation sections are meant to be a practical starting point to orient readers to concepts and ways to seek further resources. We do not comment on the success or sustainability of any method and refer the reader to resources, including tools and practical tactics, to help with implementation efforts.

## Methods

This article was researched and written by representatives from each Compendium author panel as well as implementation and healthcare epidemiology subject-matter experts, Dr. Kavita Trivedi and Joshua Schaffzin. Unlike the HAI-prevention articles in the “Compendium of Strategies to Prevent HAIs in Acute Care Hospitals: 2022 Updates,” this Compendium article is not based on a systematic literature search specific to its topic. Instead, the overview of implementation and selection of models, frameworks, and resources is based on implementation articles identified through (1) the systematic literature reviews conducted for each HAI-prevention Compendium section, (2) expert opinion and consensus, (3) practical experience, and (4) published research and resources retrieved by the authors.

Rather than providing practice recommendations, a sample of implementation models and frameworks is provided, selected for their track records in published research, utility in advancing infection prevention and control goals, and/or widespread or broad-based applicability relevant to infection prevention and control aims. A glossary of terms relevant to implementation methodology is also provided.

This document was drafted via email correspondence and video conferences among the authors, and its content was approved by electronic vote. The Compendium Expert Panel of members, with broad healthcare epidemiology and infection prevention expertise, reviewed the draft manuscript. Following review by the Expert Panel, the 5 Compendium Partner organizations, professional organizations with subject-matter expertise, and CDC reviewed the document and submitted comments. After revisions by the authors, it was reviewed and approved by the SHEA Guidelines Committee, the Infectious Diseases Society of America (IDSA) Standards and Practice Guidelines Committee, the American Hospital Association (AHA), and The Joint Commission, and the Boards of SHEA, IDSA, and the Association for Professionals in Infection Control and Epidemiology (APIC).

All panel members complied with SHEA and IDSA policies on conflict-of-interest disclosure.

## Rationale and statements of concern

The fields of infection prevention and healthcare epidemiology protect patients and the healthcare personnel (HCP) who care for them from HAIs and other safety risks through evidence-based best practices to improve population health and safety.^1^ Sustained infection prevention relies on lasting adherence to these practices to achieve desired outcomes, accountability in the process, and the application of methodologies to monitor and evaluate knowledge and performance. Regulatory authorities like the Centers for Medicaid and Medicare Services and the Occupational Safety and Health Administration,^2,3^ as well as accrediting organizations like The Joint Commission^4^ and DNV,^5^ require implementation of organizational policies and stated practices, which they have incorporated into survey expectations.^6,7^

Eccles and Mittman^8^ define implementation science as “the scientific study of methods to promote the systematic uptake of research findings and other evidence-based practices into routine practice.” Implementation science emerged in the last 20 years to improve patient outcomes and HCP safety.^9,10^ As a field of study initially developed for industry, its principles have been adapted to integrate evidence-based practices sustainably in healthcare settings. Implementation science identifies generalizable methods and frameworks to increase the utilization of evidence-based interventions deliberately and systematically in healthcare. Various terms have been used to describe the field of implementation science, including the ‘theory-practice gap,’ ‘knowledge transfer,’ and ‘knowledge utilization.’^11^ Simply put, implementation science provides the tools and frameworks to help translate evidence-based interventions into everyday clinical practice.

Studies in implementation science make it clear that identifying effective interventions is a necessary first step and that transferring them into real-world settings requires an intentional process. Education and training have proven necessary but insufficient for improvement and behavior change. Implementation science directs us to evaluate contextual determinants of behavior to design more successful, customized interventions. Improvement science, a related field, focuses on the local context and provides guidance regarding how to perform trials of new practices rapidly and iteratively to improve care.^12^ These two fields, while having distinct models and terminology, can be aligned and complement each other to improve healthcare services.^12^

HCP and teams often are unable or unprepared to implement best practices given the idiosyncrasies and complexities of healthcare settings.^13^ Identification and application of multifaceted strategies are necessary to ensure progress toward improvement.^14,15^

## Strategies for implementation

### Determinants

Foundational to any implementation effort is understanding factors that promote or hinder change. Promoting factors are called ‘facilitators’ and hindering factors are ‘barriers.’ Determinants of these factors may be individual, such as the preferences, needs, attitudes, and knowledge of HCP, hospital leaders, patients, and visitors. An individual may be a strong, engaged leader (a facilitator) or an unengaged obstructor (barrier). Determinants may include a team’s composition or ways of communicating, an organization’s culture and capacity, or a system’s policies and resources.^16^ Organizationally, implementation may be facilitated or impeded by expectations and allocation of time (eg, competing priorities, data collection burden, provision of time to dedicate to an effort, fast turnaround at the expense of sustained processes), resources (eg, ease of adapting the EMR, staff capacity, and turnover), and leadership support^17^ or follower buy-in.^18^

Facilitators and barriers affect implementation to differing degrees. For example, an individual practitioner may oppose a change (ie, be a barrier), but the supervisor may be able to facilitate to overcome the opposition. Alternatively, a practitioner may champion a change, but without the support of the leadership, they may be unable to initiate it. Additional influential factors include context, level of engagement, and reliability (see Table 1 for a glossary of terms).

Failure by HCP to adhere to a guideline or standard is a common basis for initiating an improvement project. The Cabana Framework^19^ is a useful tool to understand how addressing real or perceived barriers can make an implementation effort successful. The framework employs 3 domains (ie, knowledge, attitude, and behavior) to understand the spectrum of barriers. The Expert Recommendations for Implementing Change (ERIC)^20^ is another resource to help map barriers to strategies and identify appropriate implementation models or frameworks.

### Measurement

Data are essential for implementation to establish baselines, identify opportunities, measure progress, and justify use of resources to organizational leaders. No single method or measure will work for all situations, and standardized measures often are not available. Different frameworks lend themselves to specific methodologies, but any chosen method must do the following:

- Be appropriate for the question(s) it seeks to answer.
- Adhere to the method’s rules for data collection and analysis. As with any project, it may be prudent to review the analytic plan with an expert to ensure that data collected will yield a result.

### Choosing measures

There are 3 general types of measures employed in implementation^21^:

1. Outcome measure: The ultimate goal of a project, such as reduced surgical site infections or improving antimicrobial susceptibility patterns.
2. Process measure: The action taken to reach the desired outcome, such as adherence to a prevention bundle or compliance with hand hygiene standards.
3. Balancing measure: An undesired outcome that could be caused by changing a system, such as increased staff absences due to dry skin from a hand hygiene product or due to side effects from a required vaccine.

Ideally, all 3 types of measures are included in a project. For example, a project seeking to reduce ventilator-associated pneumonia (VAP, ie, outcome) seeks to increase early extubation (ie, process) but needs to ensure a rise in reintubations or unplanned intubations is not occurring (ie, balancing). At times, a balancing measure may be difficult to identify due to the rarity of an event or an indirect relationship between outcome or process and balancing measures. In the VAP example, using the rate of nonventilator hospital-acquired pneumonia (NV-HAP) could help identify patients who develop NV-HAP following extubation (implying they were extubated too early), but not every patient with NV-HAP will have been intubated prior to diagnosis.

Similarly, choosing an outcome measure may be difficult if the likelihood of an outcome is multifactorial or exceedingly rare. In the case of hand hygiene, improved adherence (process) should prevent nosocomial transmission (outcome). However, an overall HAI rate may not reflect the change because hand hygiene is one of many potential factors that affect nosocomial transmission. Also, it may not be possible to count all prevented transmissions. For example, a patient would not be counted if they experienced onset of an upper respiratory infection following discharge but did not require readmission. In the case of antimicrobial resistance, improved contact precaution adherence for multidrug-resistant organism (MDRO) patients (ie, process) and improved antimicrobial stewardship (ie, process) should decrease morbidity and mortality due to antimicrobial resistance (ie, outcome) but may be difficult to demonstrate in a single center or short period.^10^ In these examples, the focus of the project might be the process and balancing measures, with attention to but not reliance on the outcome.

Often, measures that are standardized and utilized broadly are referred to as ‘benchmarks.’ Measures also may be developed locally and used in a combination with benchmark measures. For instance, a facility may start with NHSN event definitions^22^ and adapt them as definitions change over time or as needed based on the suitability to their setting (eg, pediatric, long-term care, home healthcare).^23^ As another example, facilities typically use theWHO 5 Moments process measures to identify occasions when HCP should perform hand hygiene during patient care, but the methods of measurement of adherence can vary. A facility may measure adherence to all moments, adherence to a specific moment, or the amount of hand hygiene product used.^24^ When possible, it is important to use the least resource-intensive means of data collection because resources are needed to feed data back to those who were monitored. Monitoring in combination with feedback has been shown to influence change and be more effective when delivered at a high frequency.^25^

### Choosing a method

Table 2 provides a nonexhaustive list of methodologies commonly used for implementation measurement. For a research-focused overview, readers are encouraged to review the 2016 SHEA series on research methods in healthcare epidemiology and antimicrobial stewardship^26^ and Livorsi et al.^10^

## Conceptual models and frameworks

The choice of implementation methods or frameworks for any given initiative relies on the context, local knowledge and experience with implementation science, and the resources available to support the effort. Numerous frameworks combine implementation principles and tools to help organizations facilitate sustainable improvements (see Table 3 for published uses and associated resources for the models and frameworks described in this document). An organization may utilize a particular implementation framework for its relevance to a specific intervention, setting, and/or need, and another for a different initiative. As a starting point when choosing a framework, an organization may review published evidence to understand what and how framework(s) were used successfully and compare them to their local context. The following additional tools can help guide selection of framework(s):

- The Consolidated Framework for Implementation Research (CFIR), which provides a repository of constructs that have been associated with effective implementation^27^
- The Expert Recommendations for Implementing Change (ERIC) process^20^ (see Table 4 for additional resources).

Frameworks may be combined or used on their own and are meant to help guide improvements through a systematic approach derived from behavioral and organizational science and research. Damschroder et al^28^ describe using CFIR to guide formative evaluations and build the implementation knowledge base across multiple studies and settings, distinguishing between descriptive implementation models and action-oriented ones.

The following models and frameworks are used in healthcare, share purposeful experimentation and evaluation to achieve sustainable change,^29^ and illustrate the variety of ways organizations may approach a problem. These models and frameworks are listed alphabetically.

### The 4 “Es”

Pronovost et al articulated the 4 Es (Engage, Educate, Execute, and Evaluate), which may be the most pervasive model used in US healthcare epidemiology.^30^ This model is well suited for large-scale projects that include multiple sites. Its cyclical nature allows for formative work and feedback to drive modifications and adaptations, and it provides a guide for resolving knowledge gaps through education. However, it does not include targeted strategies to address multilevel barriers that hinder putting knowledge into practice.

The following 4 E strategies guide organizational change efforts:

1. Engagement: To motivate key working partners to take ownership and support the proposed interventions.
2. Education: To ensure key working partners understand why the proposed interventions are important.
3. Execution: To embed the intervention into standardized care processes.
4. Evaluation: To understand whether the intervention is successful.

The 4Es guide improvement teams in planning to address key partners for the implementation process: senior hospital leaders, improvement team leaders, and frontline staff. Planning for and utilization of multifaceted interventions that address the 4Es, coupled with explicit efforts to improve teamwork and safety culture,^31^ have been associated with reductions in HAIs^32–34^ and mortality^35^ and increased cost savings.^36^

### Behavior Change Wheel

The Behavior Change Wheel (BCW) is the result of an effort to link interventions with targeted behaviors more directly. It was developed by Michie et al,^37^ who evaluated 19 existing behavior change frameworks for comprehensiveness (ie, applicability to any intervention), coherence, and link to a behavioral model. The result was a 3-layered tool:

1. A behavior system composed of capability, opportunity, and motivation (COM-B)
2. Nine intervention functions that can be used to affect behavioral change
3. Seven policy categories that enable or support interventions to enact the desired behavior change.

One strength of the model is its nonlinearity, meaning that >1 behavioral system component, intervention function, and policy category can apply to an effort to affect change. Additionally, the model attempts to incorporate contextual influences on behavior, which the authors refer to as ‘automatic’ functions such as emotions and impulses that arise from associative learning and/or innate dispositions, as opposed to more reflective processes involving evaluations and plans. The BCW has been used widely in health promotion efforts such as smoking cessation^38^ and obesity and sedentary behavior reduction,^39–41^ and COM-B has been used to investigate hand hygiene adherence^42,43^ and antibiotic prescribing.^44,45^

### Comprehensive Unit-based Safety Program (CUSP)

CUSP focuses broadly on the idea of safety culture by empowering HCP to take responsibility for safety in their area, rather than defining specific domains of impact. As described by Pronovost et al^46^ at Johns Hopkins, who developed and validated the CUSP model in intensive care settings in 2005, the program is composed of 8 steps:

1. Culture of safety assessment
2. Sciences of safety education
3. Staff identification of safety concerns
4. Senior executive adoption of a unit
5. Improvements implemented from safety concerns
6. Documentation and analysis of efforts
7. Sharing of results
8. Culture reassessment.

The US Agency for Healthcare Research & Quality (AHRQ), the federal agency that provided funding for the development of CUSP, maintains an updated version of this framework on its website.^33,47^ The AHRQ also funded “On the CUSP: Stop CAUTI,” a national program to reduce the incidence of CAUTI through technical and hospital culture adaptations rooted in the CUSP model.^48^ This program focused on culture change.^49^ CUSP has also been used in the ambulatory setting, specifically in the AHRQ “Safety Program for Improving Antibiotic Use,” a program derived from CUSP model concepts, designed to reduce overprescribing of antibiotics in primary care.^50^ The AHRQ has further extended CUSP into ambulatory surgery, providing a full toolkit for the prevention of surgical-site infection on their website.^51^ Investigators and implementation scientists have continued to use the CUSP approach in a variety of clinical settings with mixed results. Although CUSP has shown success in preventing HAIs, such as CLABSI in US intensive care units^32,52^ and CAUTI on medical-surgical floors in acute-care hospitals^53^ and in nursing homes,^54^ not all interventions using a CUSP-based approach have been successful.^55^

### European and Mixed Methods

The European and Mixed-Methods framework derives from the CFIR^27^ and originated as the ‘InDepth’ work package,^56^ a longitudinal qualitative comparative case study within the Prevention of Hospital Infections by Intervention and Training (PROHIBIT) study.^57,58^ Specifically, InDepth sought to identify the role contextual factors play in barriers and facilitators to successful implementation.^59^ The framework defines 3 qualitative measures of implementation success:

1. Acceptability: Satisfaction with the intervention.
2. Intervention fidelity: Local implementation matched with the stated goals of the multicenter trial.
3. Adaptation: Local efforts to match the intervention with local context.

The framework has not been applied beyond the PROHIBIT outcomes of CLABSI rates and hand hygiene adherence,^57,58^ but the reported results may be used to inform other approaches.

### Getting to Outcomes (GTO)^®^

Getting to Outcomes (GTO)^®^ is a means of planning, implementing, and evaluating programs and initiatives developed for community settings. GTO^®^ seeks to build capacity for self-efficacy, attitudes, and behaviors to yield effective prevention practices.^60^ The process involves 10 steps:

- Steps 1–5: Assess and evaluate needs, goals, and feasibility of a proposed program.
- Step 6: Plan and deliver the program.
- Steps 7–10: Evaluate, improve, and sustain successes.

GTO^®^ has been utilized for numerous community-based initiatives, such as evidence-based sexual health promotion,^61^ a dual-disorder treatment program for veterans,^62^ and development of casework models for child welfare services.^63^ Additionally, the RAND Corporation has published a guide to develop community emergency preparedness programs. This guide breaks down each of the 10 GTO^®^ steps, provides materials and examples,^64^ and may facilitate the use of GTO^®^ in implementing infection prevention interventions.

### Model for Improvement

The Model for Improvement^65^ was developed by the Associates for Process Improvement based on Deming’s System of Profound Knowledge.^66^ It has since been adopted widely, perhaps most notably by the Institute for Healthcare Improvement (IHI) in its 100,000 and 5 Million Lives campaigns of the early 2000s.^67^ The model has been used to accelerate change in a variety of healthcare and public health settings,^68–70^ and subspecialists have created primers focused on their practice areas.^71–74^ The Model for Improvement begins with 3 questions:

1. What are we trying to accomplish?
2. How will we know that a change is an improvement?
3. What change can we make that will result in improvement?
  Once those questions are answered, the identified changes or interventions are tested using plan–do–study–act (PDSA) cycles. Individual tests include the following:

- Plan: Predictions of outcome.
- Do: Executed according to plan.
- Study: Analysis and evaluation.
- Act: Decision whether to keep, abandon, or modify the intervention.
  PDSA findings and decisions then guide planning the next experiment, starting a new PDSA cycle. Multiple cycles are done in series called ‘ramps.’ The Model for Improvement is designed for team-driven projects, relies heavily on data analysis and interpretation, and requires training (much of which can be self-directed online).

### Reach, Effectiveness, Adoption, Implementation, Maintenance (RE-AIM)

Reach, effectiveness, adoption, implementation, and maintenance make up the 5 dimensions of the planning and evaluation framework RE-AIM, developed to address the failures and delays in translating scientific evidence into policy and practice.^75,76^ By utilizing these 5 dimensions at individual and ecological levels, teams can better understand the effectiveness of programs as they are implemented in real-world community settings.^77,78^

RE-AIM is useful for planning an intervention, the outcomes that will be measured, and evaluating whether the intervention has met its goals.^79^ All 5 dimensions are not always addressed. In recent years there has been greater emphasis on pragmatic application of the framework to determine which dimensions an organization should prioritize.^80^ It also provides ideas for quantitative measurements of outcomes.

RE-AIM was utilized to evaluate an antimicrobial stewardship program in an ICU in South Africa,^81^ dissemination and implementation of clinical practice guidelines for sexually transmitted infections,^82^ and promotion of vaccination via digital technology.^83^ Recently, to better understand the implementation of contact tracing for emerging infectious diseases, RE-AIM was used to evaluate individual and systems-level predictors of success of an emergency volunteer COVID-19 contact tracing program in Connecticut.^76,78,84^ Investigators concluded that the program fell short of CDC benchmarks for time and yield, largely due to difficulty collecting the information necessary for outreach.

### Replicating Effective Practices (REP)

The Replicating Effective Programs (REP) framework may be used to balance needs of the target population with the core elements of successfully implemented interventions^85^ and to maximize fidelity to core interventions that have been rigorously tested and have produced statistically significant positive results.^86^

There are 4 phases of REP^87^:

1. Preconditions (ie, identification of needs)
2. Preimplementation (eg, community input)
3. Implementation (eg, training)
4. Maintenance (eg, preparing for sustainability).

REP may be useful when adapting interventions for a specified target audience within healthcare. It also may be applied across the continuum of care (eg, acute care to long-term care) or in multifacility systems when local institutional culture dictate the need for adaptation. When used to disseminate evidence-based HIV prevention interventions to community-based organizations, the application of REP to packaging, HCP training, and technical assistance resulted in more effective uptake than dissemination alone.^88^

### Theoretical domains

The Theoretical Domains Framework (TDF) was initially developed to conduct research on the behavior of HCP as it relates to implementing evidence-based practices.^89^ The initial organization of TDF^90^ was modified following a formal validation exercise,^91^ which yielded 14 domains to identify relevant cognitive, affective, social, and environmental influences on behavior.^89^ TDF has been used widely to understand and influence HCP,^92^ patient, and population behaviors, most commonly with qualitative methods (eg, surveys, interviews, and/or focus groups).^89^ One salient example is a patient safety effort to properly place nasogastric tubes. A team utilized a validated TDF-based questionnaire to identify relevant domains that were then explored in focus groups to help connect theory to techniques to change behavior.^93^ More recently, TDF was used to develop the Choosing Wisely De-Implementation Framework^94^ that proposes to reduce low-value care, defined as a test or treatment for which there is no evidence of patient benefit or where there is evidence of more harm than benefit.^95^ A guide to TDF use^89^ and a 4-step systematic approach to using TDF^96^ were published to help teams design and follow through with an intervention. TDF has been linked to the COM-B model (used in the aforementioned BCW) and has been used in combination with other frameworks when the time necessary to complete interviews and focus groups was limited.^97^

## Future needs

### Models for underperforming hospitals and units

Allthough national implementation studies have succeeded in preventing several different HAIs, investigators have not seen the same success when focused on facilities most in need of help—hospitals underperforming with respect to HAI prevention. The CDC-funded national prospective, interventional, quality improvement program, CDC STRIVE (States Targeting Reduction in Infections Via Engagement), focused on reducing CLABSIs, CAUTIs, *Clostridioides difficile* infections, and methicillin-resistant *Staphylococcus aureus* (MRSA) bloodstream infections in hospitals with a disproportionately high burden of HAIs.^98^ Although nearly 400 US hospitals participated in this multimodal, multifaceted, partner-facilitated program, they did not see significantly reduced rates of CLABSI,^99^ CAUTI,^100^ C *difficile* infection,^101^ or MRSA bloodstream infection.^102^ More recently, the Agency for Healthcare Research and Quality (AHRQ) funded a national program that invited US hospitals that had at least 1 adult ICU with elevated CLABSI or CAUTI rates to participate in an externally facilitated program implemented by a national project team and state hospital associations using the Comprehensive Unit-based Safety Program (CUSP) framework.^103^ Results from the first 2 cohorts (366 recruited ICUs from 220 hospitals in 16 states and Puerto Rico) revealed no statistically significant reductions in CLABSI, CAUTI, or catheter utilization in the 280 ICUs that completed the program.^103^ These researchers cite a number of possible factors contributing to the disappointing result, including underutilization of training and coaching resources, lack of infrastructure, and a different selection process for participants (eg, identifying low-performing units or those that had been unsuccessful to date versus asking for volunteers for earlier CUSP work, which may have selected for early adopters and high performers). Investigations in this and similar cohorts could help elucidate why hospitals with disproportionately high HAI rates have not yet seen significant reductions in HAIs despite broad-based efforts. Increased focus on the development, adaptation, and utilization of implementation models and frameworks to infection prevention and control may help identify implementation gaps that contribute to lack of improvement and guide their closure.

### Sustaining system change

A long-term goal of any implementation effort is to sustain and advance short-term gains. Ideally, sustaining gains occurs with less intensity than initial efforts, maintaining gains or improving at a slower rate and allowing resources to be directed to another effort. Characteristics of successfully sustained interventions have included those that are incorporated into the standard workflow, have effective champions to shepherd the effort and re-engage when necessary, can be modified over time, fit with an organization’s mission and procedures, provide easily perceived benefits to staff members and/or clients, and are supported by partner organizations.^104^ It can be difficult to meet those criteria in healthcare, where changes in workflows and staff are frequent.^105^ Demonstrating successfully sustained implementation should include evidence of (1) sustainment, that is, sustained use of an evidence-based intervention (process measure), and (2) sustainability, that is, sustained benefits of an evidence-based intervention (outcome measure).^106^

Linking ongoing process data to ongoing outcome data can prove challenging. In one study, CLABSI reduction in ICUs was sustained for a decade, but process measurement was not performed.^34^ A study on CAUTI prevention at a Veterans’ Affairs (VA) hospital found that, 8 years after implementation, appropriateness of urinary catheters remained high and stable.^105^ Catheter use decreased, but the facility was unable to report outcome data.^105^ These researchers hypothesized that success was due to a 3-component, evidence-based intervention, institutionalization of the interventions (ie, standardizing nursing assessments and handoffs that included the intervention), and effective champions who re-engaged when necessary. Additionally, a study on hand hygiene on 2 hospital units in Italy found that adherence dropped after 4 years (from 84.2% to 71%) despite maintaining champions and processes.^107^ Recent proposals for standard definitions^106,108^ and modified ERIC strategies to account for sustainment and identify interventions that yield short-term and sustained improvements^109^ can form a basis for future research and understanding.

### Conclusion

It is increasingly evident that implementation is essential to ensuring that evidence-based interventions are performed to generate desired outcomes and to meet infection prevention and control and antimicrobial stewardship goals.^10^ Furthermore, a detailed implementation plan in a specific healthcare setting for a given intervention is necessary for success, as the implementation approach in one facility may not be reproducible, with the desired effect, in another. In this article we have provided an overview of implementation in a general sense, with a glossary of terms, broader discussion of key methods, models, and frameworks, possible future areas of study, as well as links to resources readers can use to initiate or continue their implementation journey.

## Supplementary Material

Supplementary Material

## Figures and Tables

**Table 1.** Glossary of Terms

Term	Definition
Implementation	• Strategies used to promote the adoption and integration of evidence-based health interventions and change practice patterns within specific settings^110^
Implementation science	• “The scientific study of methods to promote the systematic uptake of research findings and other evidence-based practices into routine practice”^8^ • Initially developed for industry and then adopted by healthcare to improve patient outcomes through deliberate, systematic, and sustained utilization of evidence-based practices • Identifies generalizable methods and frameworks • For a discussion of implementation science in antimicrobial stewardship, see Livorsi et al.^10^
Improvement science	• Behaviorally focused guidance for how to trial practices rapidly and iteratively to improve care based on the local context^12^ • Acknowledges that improvement is a continually evolving process, requiring adjustments and reinforcements to achieve success • Related to implementation science, but with distinct models and terminology • Implementation science and improvement science can be aligned to complement each other to improve healthcare services.^12^
Quality improvement	• Use of a deliberate and defined improvement process to achieve measurable improvements in efficiency, effectiveness, performance, accountability, outcomes, and other indicators of quality in services^111^ • Continuous, ongoing effort to enhance patient outcomes and experiences, reduce the cost of healthcare, and improve the HCP experience^112^
Acceptability	• Satisfaction with the intervention (European and Mixed Methods Model)
Accountability	• Defined as system, collective, and individual ownership of results • Important for being able to evaluate practice deviations • Applies to the microsystem, mesosystem, and macrosystem: • Punitive actions toward individuals have been shown to be less effective.^66^ System, not individual performance, should be measured to facilitate shared solutions. • Accountability depends on a supportive healthcare infrastructure. • HCP should have clear and realistic expectations for how to adhere to best practices.^113^
Adaptation	• Efforts to match the intervention to local context (European and Mixed-Methods Model)
Apparent cause analysis	• A process used to examine a safety event or near miss^114^ • Can guide future efforts by identifying why a success or failure occurred • When failures persist or an apparent cause cannot be identified, process mapping and direct observations with staff (‘walking the process’) may help gain insight into unidentified barriers.^30,52,115,116^
Balancing measure	• An undesired outcome that could be caused by changing a system, such as increased staff absences due to dry skin from a hand hygiene product or due to side effects from a required vaccine
Barrier	• Hindering factor
Buy-in	• Acceptance of and willingness to actively support and participate in proposed new practices or policies^114^
Champions	• Trusted persons who directly or indirectly are involved in shepherding a decision or intervention^117^ and: • Know their hospital’s interests and needs • Have the ability to gain buy-in to shape strategies to match local unit culture, monitor progress, and facilitate necessary changes during implementation^52,116,118,119^ • Can engage HCP to answer questions, resolve concerns, prepare for action, and sustain improvements^116,118,120^
Context	• Local factors such as operational support, informatics resources, familiarity and experience, willingness to change, safety culture, etc. that impact an implementation effort. Context may encapsulate setting, healthcare workforce, patient population, and the specific practice or intervention.
Dissemination	• Targeted distribution of information and intervention materials to a specific public health or clinical practice audience^110^
Education	• Support consistency in adoption of new practices, setting of expectations, fidelity to the evidence, and buy-in^118,121–126^ • Potential education participants: • HCP and staff • Patients • Caregivers and family members, to help them adhere to safe practices and to support the plan of care^127, 128^ • Multifaceted resources: • Information sharing (see Peer networks) • Online and in-person training opportunities • Simulations • Materials for self-study^129–134^ • Education alone is insufficient for successful implementation and should not be relied on as a sole approach for longterm sustainability^135^
Empowerment	• Access to support, resources, information, and opportunities to learn and grow. Characterized by collaborative relationships and autonomy in decision making^136^
Engagement	• Personal engagement: An intention of individuals to bring their best self to work, grow personally and professionally in their work roles, and contribute to the organization through their thoughts, feeling, and physical energies. • Job engagement: A positive, fulfilling work-related attitude characterized by high resilience, intense effort, and focus.^137^
Facilitator	• Promoting factor
Feedback	• A summary of performance within a specified period^25^ • Visibility of system-level data is important to achieve and sustain change by: • Establishing expectations and creating urgency • Assuring working partners’ ongoing, real-time awareness of status • Rapidly identifying issues • Providing insight into problems, and ways to target interventions to avoid them^30,52,123,138,139^ • Maintaining team motivation by helping them visualize success • Rewarding and solidifying behavior changes • Marking successful transitions to new standards of care^30,52,115,122,140-142^
Fidelity	• The extent to which the practice remains true to the evidence • Local implementation matched to the stated goals of the multicenter trial (European and Mixed-Methods Model)
Human factors engineering (HFE)	• When designing and testing interventions, HFE is a process for accounting how people interact with technology, each other, and the environment^29^ • HFE principles that are particularly relevant in healthcare: • Orientation to a system, rather than an individual • Supporting work with systems that fit capabilities, needs, and limitations of working partners • Identifying outcomes that are achievable through intentional design^143^ • Standardizing practice through the use of dedicated teams, informatics, and/or checklists to establish processes as “normal behaviors”
Implementation team	• Team-based approach: • Underpinned by a shared mental model around a problem to be solved • Dedicated to developing and mobilizing around a solution • Includes representatives of key working partners, and may also include: • Hospital and system leadership to help give the effort legitimacy, demonstrate commitment, remove barriers, and sustain improvements. Leaders may be in formal (eg, medical director, nursing director, charge nurses, healthcare executives) and informal (eg, influential frontline staff) roles • Experts beyond target group (eg, subject matter, improvement science, data analysis, information technology) • Local champions • Patient or caregiver advocates^127,144^ • Team responsibilities^30,120,144-152^ include: • Development of a shared mental model with commitment to the identified solution • Identification of: • Touch points for the practice or intervention • Who to engage • How to engage them • Determinants (barriers and facilitators to the uptake of the practice or intervention) • Implementation plan development and deployment • Dissemination • Measurement and feedback
Measurement and monitoring	• Measurement of practices should occur based on datapoints and design metrics identified for their ability to inform priorities, lead to action, and be made visible • Data collection and auditing should be purposeful, focused, efficient, and consistent • Can be done using frequent formal and informal audits of clinical practice^30,118,123,126,127,133,140,153-156^ or via automated monitoring to alleviate the burden of manual chart review or observation • The value of individual monitoring and real-time feedback can be beneficial for complex processes^157,158^
Outcome measure	• The ultimate goal of a project, such as reduced surgical-site infections
Peer networks	• Voluntary hospital, system, or local healthcare personnel networks • Support collaboration for change through shared awareness of and investment in local scenarios and epidemiology • Peer networks can: • Encourage collaboration, analysis of performance, accountability, and commitment to specific goals^123,126,131,139,144,159,160^ • Compare progress and set benchmarks to help groups understand their strengths and weaknesses, learn from best practices, brainstorm solutions to common problems, and promulgate success^119,123,126,128,131,139^ • May focus on one topic, eg, preventing a particular HAI, or different topics with a focus on sharing strategies to make interventions acceptable, feasible, and sustainable, and ways to approach engagement, buy-in, accountability, education, measurement
Process mapping	• A written-out, algorithm-like map of how a process functions • Enhances understanding of an existing system, identifies issues, and helps organizations and teams plan interventions • Can facilitate multidisciplinary understanding by providing a visual representation of an improvement effort^161^ • Involves minimal expertise and can be especially effective in resource-constrained settings^162^
Process measure	• The action taken to reach the desired outcome, such as adherence to a prevention bundle or compliance with hand hygiene standards
Reflective motivation	• Collaborative technique for eliciting positive or negative feelings about adoption of a new practice^15^ to increase knowledge, understanding, and commitment among a multidisciplinary group
Reliability	• The frequency at which an intervention is completed when indicated • Reliability can be supported by: • Standardization to facilitate system evaluation and improvement efforts^118,119,122,126,132,139,151,153^ • Clinical informatics to streamline clinical workflows and develop reports and analytics for problem recognition and resolution
Reminders	• Visual and verbal reminders can support education and increase the likelihood that best practices are followed.^30^,^118^,^121^,^127^,^132^,^141^,^153^,^163^-^167^ • Over time, visual reminders (eg, posters, bulletins, signs, daily goal lists, checklists, screensavers) may become less effective at holding their targets’ awareness.^168^ • The benefit of certain types of reminders, such as point-of-care alerts, is typically small.^169^ • Visual reminders can create clutter and lead to reminder fatigue over time.^170^ • Visual reminders should be used judiciously, updated, and cycled to maintain working partners’ awareness of them. • Multidisciplinary huddles or rounds can be an effective way to reinforce reminders. • Include discussion about goals for the day, resources and actions needed, and potential barriers or safety issues. • Follow a structured format. • Provide verbal reinforcement of expectations. • Foster good habits among the process participants.^127,144,148,156,171-174^
Surveillance	• Ongoing systematic collection, analysis, and interpretation of health-related data essential to planning, implementation and evaluations of public health practice^175^
Surveillance and improvement networks	• Collect and supply data or directly engage organizations and HCP to learn collectively • Exist in the United States and internationally • Examples: Solutions for Patient Safety, the National Surgical Quality Improvement Program (NSQIP), and statewide collaboratives
Sustainability	• A domain of quality improvement that takes a long-term perspective on the needed support, resources, and return on investment related to maintaining a practice or intervention for patients today and in the future^176^; sustained benefits of an evidence-based intervention (outcome)^106^
Sustainment	• Sustained use of an evidence-based intervention (process)
Translation	• Closing of gaps between knowledge or evidence and practice. • The US National Institutes of Health (NIH) defines translation as, “the process of turning observations in the laboratory, clinic and community into interventions that improve the health of individuals and the public.” This includes diagnostics, therapeutics, medical procedures and behavioral changes.^177^ • Knowledge translation is defined by the Canadian Institutes of Health Research as “a dynamic and iterative process that includes the synthesis, dissemination, exchange and ethically sound application of knowledge to improve health, provide more effective health service and products, and strengthen the healthcare system.”^178^
Working partners	• Persons or groups who may be affected directly or tangentially by proposed changes to workflow, practice, or hospital structure due to implementation of practice or intervention • May include: • Individuals and groups • Personnel in clinical and nonclinical roles • Hospital and system leaders • Caregivers and family members • Front desk staff • Environmental services • Medical assistants • Supply chain staff

**Table 2.** Methods for Measurement

Method	Input Data	Output	Frameworks and Examples	Notes
Statistical Process Control	Events per period	Mean or median event rate/occurrence	Model for Improvement Lean/Six Sigma	Based on identifying deviations from a baseline value, requires construction of event definition, access to data (eg, patient days, vascular catheter days, etc)
Qualitative	Interviews, focus-group transcripts, testimonials	Themes related to beliefs, practices, perceptions	Theoretical Domains Replicating Effective Practices	Can help guide project target/focus Can test and postintervention effectiveness Open-ended allows probing but more labor intensive to analyze
Surveys	Answers to questions on an existing or de novo survey	Descriptive data (practices) Association testing	Behavior Change Wheel/COM-B Theoretical Domains	Subject to pitfalls and bias
Delphi	Degree of agreement with statements or practices	Multiple rounds of ranking and feedback of results until consensus reached	Bright STAR, used for quality improvement/implementation	An organized method to reach consensus for practices for which high quality evidence lacks
Mixed Methods	Qualitative and quantitative or survey data			

**Table 3.** Implementation Frameworks

Framework	Published Experience	Resources
4Es	**Settings** • Healthcare facilities • Large-scale projects including multiple sites **Infection prevention and control** • HAI Prevention (including mortality reduction and cost savings)	• 4Es framework^30^ • HAI reduction^32-34^ • Mortality reduction^35^ • Cost savings^36^
Behavior Change Wheel	**Settings** • Community-based practice • Healthcare facilities **Healthy behaviors** • Smoking cessation • Obesity prevention • Increased physical activity **Infection prevention and control** • Hand hygiene adherence • Antibiotic prescribing^179^	• Behavior Change Wheel: A Guide to Designing Interventions • Stand More at Work (SMART Work)^41^
CUSP	**Settings** • Intensive care units • Ambulatory centers **Improvements** • Antibiotic prescribing • CLABSI prevention • CAUTI prevention	• CUSP Implementation Toolkit • AHA/HRET: Eliminating CAUTI (Stop CAUTI) • AHRQ Toolkit to Improve Safety in Ambulatory Surgery Centers
European Mixed Methods	**Settings** • European institutions of varied healthcare systems and cultures **Improvements** • CLABSI prevention • Hand hygiene	• PROHIBIT: Description and Materials
Getting to Outcomes (GTO)	**Settings** • Community programs and services **Improvements** • Sexual health promotion • Dual disorder treatment program in veterans • Community emergency preparedness	• RAND Guide for Emergency Preparedness (illustrated overview of GTO methodology)
Model for Improvement	**Settings** • Healthcare (inpatient, perioperative, ambulatory), public health **Interventions** • PPE use • HAI prevention • Public health process evaluation	• Institute for Healthcare Improvement • The Improvement Guide • Deming’s System of Profound Knowledge
Reach, Effectiveness, Adoption, Implementation, Maintenance (RE-AIM)	**Settings** • Healthcare • Public health • Community programs • Sexual health **Evaluation** • Antimicrobial stewardship in ICU • Clinical practice guidelines for STIs • Promotion of vaccination • Implementation of contact tracing	• RE-AIM.org • Understanding and applying the RE-AIM framework: Clarifications and resources^80^
Replicating Effective Practices (REP)	**Settings** • Healthcare • Public health • HIV prevention • Interventions that have produced positive results are reframed for local relevance	• CDC Compendium of HIV Prevention Interventions with Evidence of Effectiveness (see Section C, Intervention Checklist)^86^
Theoretical Domains	**Settings** • Healthcare (inpatient, perioperative, ambulatory) • Community (individual and community-based behaviors) **Health maintenance** • Diabetes management in primary care • Pregnancy weight management **HCP practice** • ICU blood transfusion • Selective GI tract decontamination • Preoperative testing • Spine imaging • Hand hygiene	• A guide to using the Theoretical Domains Framework of behavior change to investigate implementation problems^89^ • Developing theory-informed behavior change interventions to implement evidence into practice: a systematic approach using the Theoretical Domains Framework^96^ • Choosing Wisely De-Implementation Framework

**Table 4.** Other Resources

Type	Resource
Websites	• Institute for Healthcare Improvement: resources including step-by-step explanation of Model for Improvement, reports on strategies and examples of projects
Databases of Models and Frameworks	• Consolidated Framework for Implementation Research (CFIR)^27^ • CFIR-ERIC Implementation Strategy Matching Tool • RE-AIM
Consultants	• Consider hiring a consultant periodically to advise your organization in implementation considerations, especially if you have limited in-house or local implementation resources. • You may seek input from fellow members of your professional organization, eg, through member forums like MySHEA and APIC’s IP Talk Digest.
Courses	• Consider supporting 1 or more HCP in an organization, or in every department, to take a course in implementation to develop in-house expertise. • NIH self-study online modules (8 modules, ~25 minutes each) • IHI Open School (online course) • Certificate of implementation science done by certain universities and elsewhere, eg, UCSF Implementation Science Program
