# Supplementary material for: Implementing strategies to prevent infections in acute-care settings
Source: Infect Control Hosp Epidemiol. Author manuscript; Available in PMC 2024 Aug 1. (PMC10527889; doi:10.1017/ice.2023.103)
Supplement: Supplementary Material [file NIHMS1922770-supplement-Supplementary_Material.docx]

# Supplementary Material

## Intervention evaluation checklist using Replicating Effective Programs (REP)

Adapted from the CDC Compendium of HIV Prevention Interventions with Evidence of Effectiveness

| **Intervention Items** | **Original Intervention** | **Local Intervention** | **Rating** |
| --- | --- | --- | --- |
| Who is the target audience? |  |  | High  Medium  Low |
| What are the goals and objectives of the intervention? |  |  | High  Medium  Low |
| What behavioral and social science theories underpin the intervention? |  |  | High  Medium  Low |
| Does the intervention focused on changing specific behaviors? |  |  | High  Medium  Low |
| Does the intervention provide opportunities to practice relevant skills? |  |  | High  Medium  Low |
| **Implementation Items** | **Original Intervention** | **Local Intervention** | **Rating** |
| Is time required to deploy the intervention realistic? |  |  | High  Medium  Low |
| Are staff adequately trained for the sensitivity to the targeted population? |  |  | High  Medium  Low |
| Are staff adequately trained to deliver the core elements of the intervention? |  |  | High  Medium  Low |
| Are core elements of the intervention clearly defined and maintained in the delivery? |  |  | High  Medium  Low |
| Do staff use a variety of teaching methods, strategies, and modalities to convey information, personalize the training and repeat essential prevention messages? |  |  | High  Medium  Low |
| **Organization Items** | **Original Intervention** | **Local Intervention** | **Rating** |
| Is administrative support for the intervention present at the highest levels? |  |  | High  Medium  Low |
| Are there sufficient resources for the current implementation? |  |  | High  Medium  Low |
| Are there sufficient resources for sustainability? |  |  | High  Medium  Low |
| Are decision-makers flexible and open to program changes |  |  | High  Medium  Low |
| Is the intervention embedded in a broader contest that is relevant to the target population |  |  | High  Medium  Low |
| **Consumer Participant Items** | **Original Intervention** | **Local Intervention** | **Rating** |
| Does the intervention meet specified priorities and needs defined by the community |  |  | High  Medium  Low |
| Is the intervention culturally appropriate for the target population selected? |  |  | High  Medium  Low |
| Is the intervention developmentally appropriate for the target population selected? |  |  | High  Medium  Low |
| Does the intervention have aspects of diversity, equity and inclusion if appropriate? |  |  | High  Medium  Low |
| Is it anticipated that when implemented the intervention will be acceptable to the participants? |  |  | High  Medium  Low |
